# A Bioactive Extract Rich in Triterpenic Acid and Polyphenols from *Olea europaea* Promotes Systemic Immunity and Protects Atlantic Salmon Smolts Against Furunculosis

**DOI:** 10.3389/fimmu.2021.737601

**Published:** 2021-11-12

**Authors:** Ricardo Salomón, M. Dolors Furones, Felipe E. Reyes-López, Lluis Tort, Joana P. Firmino, M. Angeles Esteban, Cristóbal Espinosa Ruíz, José C. Quintela, José M. Pinilla-Rosas, Eva Vallejos-Vidal, Enric Gisbert

**Affiliations:** ^1^ Aquaculture Program, Institut de Recerca i Tecnologia Agroalimentàries (IRTA), Centre de Sant Carles de la Ràpita (IRTA-SCR), Sant Carles de la Ràpita, Spain; ^2^ PhD Program in Aquaculture, Universitat Autònoma de Barcelona, Bellaterra, Spain; ^3^ Department of Cell Biology, Physiology and Immunology, Universitat Autònoma de Barcelona, Bellaterra, Spain; ^4^ Facultad de Medicina Veterinaria y Agronomía, Universidad de Las Américas, Santiago, Chile; ^5^ Centro de Biotecnología Acuícola, Departamento de Biología, Facultad de Química y Biología, Universidad de Santiago de Chile, Santiago, Chile; ^6^ Department of Cell Biology and Histology, Faculty of Biology, University of Murcia, Murcia, Spain; ^7^ Scientific Department, Natac Biotech, Alcorcón, Madrid, Spain

**Keywords:** feed additive, *Olea europaea*, aquaculture, *Aeromonas salmonicida*, challenge, systemic immunity, *Salmo salar*, immune homeostasis

## Abstract

In the present study, the modulation of the transcriptional immune response (microarray analysis) in the head kidney (HK) of the anadromous fish Atlantic salmon (*Salmo salar*) fed a diet supplemented with an olive fruit extract (AQUOLIVE^®^) was evaluated. At the end of the trial (133 days), in order to investigate the immunomodulatory properties of the phytogenic tested against a bacterial infection, an *in vivo* challenge with *Aeromonas salmonicida* was performed. A total number of 1,027 differentially expressed genes (DEGs) (805 up- and 222 downregulated) were found when comparing the transcriptomic profiling of the HK from fish fed the control and AQUOLIVE^®^ diets. The HK transcripteractome revealed an expression profile that mainly favored biological processes related to immunity. Particularly, the signaling of i-kappa B kinase/NF-kappa and the activation of leukocytes, such as granulocytes and neutrophils degranulation, were suggested to be the primary actors of the innate immune response promoted by the tested functional feed additive in the HK. Moreover, the bacterial challenge with *A*. *salmonicida* that lasted 12 days showed that the cumulative survival was higher in fish fed the AQUOLIVE^®^ diet (96.9 ± 6.4%) than the control group (60.7 ± 13.5%). These results indicate that the dietary supplementation of AQUOLIVE^®^ at the level of 0.15% enhanced the systemic immune response and reduced the *A*. *salmonicida* cumulative mortality in Atlantic salmon smolts.

## 1 Introduction

The worldwide production of farmed Atlantic salmon (*Salmo salar*) has progressively increased from 294 t in its inception in 1970 up to 2,615,962.4 t in 2019, with Norway and Chile being the main producers with 1,364,042 t (52.1%) and 701,731 t (26.9%), respectively (1). This flourishing industry has grown focusing their efforts on profitability, competitiveness, and sustainable development; however, disease is the biggest risk to the industry, since it undermines financing and market development. In particular, infectious diseases represent a major problem in worldwide salmon farming, despite the successful development and application of vaccines against a wide range of pathogens and the implementation of management practices for fighting against parasites (2). In this sense, intensified production systems and climate change will favor the occurrence of disease outbreaks due to the farming of more stressed and immuno-compromised animals in farms, and the evolution and spread of more virulent pathogens. This qualifies aquatic animal diseases as one of the major limiting factors for aquaculture development (3, 4).

Although in recent years, there has been a drastic reduction in antibiotic use in some countries due to vaccination and improved husbandry practices, the use of antimicrobials is still a common practice in order to avoid and mitigate potential production and economic losses derived from outbreaks of pathogenic organisms (5, 6). In this sense, the academy and the industry have merged efforts in order to develop, test, and validate sustainable and environmentally friendly alternative treatments in order to prevent disease outbreaks and to reduce the use of chemotherapeutic drugs. Among the repertoire of tested strategies (7, 8), functional feeds are considered as one of the most affordable and sustainable preventive solutions (9). Feeds that provide physiological benefits beyond the animal’s basic nutritional requirements are named as functional feeds, and their use has progressively gained attention within the aquaculture. Feed additives may be divided into different categories considering the purpose of their use (nutritional, sensorial, and functional additives), which also affects their chemical nature and mode of action (10, 11). In this sense, functional feed additives with immunomodulatory properties and capacity of relieving stress and promoting disease resistance in farmed animals are of interest as sustainable health management strategies. The most widely evaluated functional feed additives, as immunostimulants, are probiotics, prebiotics, symbiotics, acidifiers, nucleotides, and phytogenics (10, 12). Among them, phytogenics are reputed for their growth-promoting effects, as well as their antimicrobial, antioxidant, anti-inflammatory, immunostimulant, and anti-stress properties (10), representing a promising effective and sustainable prophylactic tool to be implemented in health management in front of bacterial and parasitic infections (13, 14).

Fruits and leaves of the olive oil tree (*Olea europaea* L.) contain significant amounts of hydrophilic and lipophilic bioactives including flavones, phenolic acids, phenolic alcohols, secoiridoids, and hydroxycinnamic acid derivates (15). As a result of their anti-inflammatory, antioxidant, and antimicrobial actions, olive-derived phytogenics have shown beneficial health effects in human (16–18) and livestock (19–21) health. However, limited information is available on their effects on aquaculture fish species (22). In pigs (20) and fish (22), an olive-oil bioactive extract, containing a mixture of triterpenic acid and polyphenols, had anti-inflammatory and immunomodulatory properties in the intestine, while it also enhanced the integrity of the epithelium. In addition, a recent study showed that these compounds were able to reduce systemic inflammation in cattle (21). Regardless of these results, little is known about the immunomodulatory effects of this olive-oil bioactive extract on the systemic immune response and its potential use as a functional feed additive in aquafeeds for promoting disease resistance in fish.

The objective of the present study was to evaluate the effects of a diet supplemented with an olive-oil bioactive extract rich in polyphenols and triterpenic acid (AQUOLIVE^®^; NATAC Biotech SL, Spain) on the systemic immune response and disease resistance in Atlantic salmon smolts. For this purpose, Atlantic salmon parrs were smoltified with a diet supplemented with AQUOLIVE^®^. The levels of several humoral immune parameters were measured and the transcriptomic profiling of the head kidney (HK) analyzed by means of a microarray, whereas the potential protection of the tested feed additive was validated by means of an *in vivo* challenge with a pathogenic bacteria (*Aeromonas salmonicida*). This bacterium was chosen because it is the causative agent of furunculosis, which has been recognized as a threat for the salmon industry, reaching mortality rates up to 50%, even though it may be controlled by the administration of antibiotics and oil-based vaccines (2). However, assessing alternative more sustainable and affordable strategies based on the administration of functional feeds is advisable.

## 2 Material and Methods

### 2.1 Diets

To evaluate the immunomodulatory properties of the phytogenic obtained from olive fruit, two isoproteic (40% crude protein), isolipid (22% crude fat), and isoenergetic (21.6 MJ/kg gross energy) diets were formulated in order to fulfill the nutritional requirements of juvenile Atlantic salmon (23). Diets named as control and AQUOLIVE^®^ were formulated to contain 17.5% fishmeal LT70, 2.5% fish protein concentrate, 55% plant-protein sources (soy protein concentrate, wheat and corn gluten faba beans, and wheat meal), and 10% fish oil and only differed in their content of the tested phytogenic (0.15%). The AQUOLIVE^®^ was obtained by NATAC Biotech SL (proximate composition: 69.23% carbohydrates, 8.19% crude lipids, 0.41% crude proteins, 9.11% salts, and 3.06% water) which contained 10% olive bioactive compounds (8.0% triterpenic acid and 2% polyphenols).

Diets were manufactured by Sparos Lda. The main ingredients were ground (below 250 μm) in a micropulverizer hammer mill (SH1; Hosokawa Micron, B.V., Doetinchem, The Netherlands). Powder ingredients and oils were then mixed according to the target formulation in a paddle mixer (RM90; Mainca, S.L., Granollers, Spain). All diets were manufactured by temperature-controlled extrusion (pellet sizes: 2 and 3 mm) by means of a low-shear extruder (P55; Italplast S.R.L., Parma, Italy). Upon extrusion, all feed batches were dried in a convection oven (OP 750-UF; LTE Scientific, Oldham, UK) for 4 h at 45°C. Samples of each diet were taken for proximate composition analysis (24) and additive quantification (information provided by the manufacturer). Feeds were stored at 4°C during the experimental period (146 days) in order to prevent their oxidation. The list of ingredients and the proximate composition of experimental diets are shown in **Table 1**.

**Table 1:** List of ingredients and proximal composition of experimental diets: control and a basal diet supplemented with AQUOLIVE^®^.

Ingredients, %	Control diet	AQUOLIVE^®^ diet
Fishmeal LT70	17.5	17.5
Soy protein concentrate	20.0	20.0
Fish protein concentrate	2.5	2.5
Wheat gluten	9.0	9.0
Corn gluten	5.0	5.0
Faba beans	5.0	5.0
Wheat meal	16.23	16.08
Fish oil	12.0	12.0
Vitamin and mineral premix	1.0	1.0
Soy lecithin	0.5	0.5
Vitamin C35%	0.07	0.07
Monocalcium phosphate	3.0	3.0
Rapeseed oil	7.0	7.0
Betaine HCI	1.0	1.0
DL-Methionine	0.2	0.2
AQUOLIVE^®^	–	0.15
**Proximate composition**		
Crude protein, %	40.03	40.02
Crude fat, %	22.15	22.15
Fiber, %	1.75	1.74
Starch, %	13.02	12.93
Ash, %	8.74	8.89
Gross Energy, MJ/kg	21.60	21.58

### 2.2 Fish and Experimental Design

A total of 1,500 unvaccinated Atlantic salmon parrs were obtained from a commercial fish farm (SARL SALMO, Gonneville-le-Thiel, France) and transported by road to IRTA-Sant Carles de la Ràpita research facilities (Sant Carles de la Ràpita, Spain). Once at IRTA facilities, fish were acclimated in two 2,000-l tanks connected to an open-flow system (water temperature: 12°C ± 1.5°C) for 2 weeks under a natural photoperiod. During the acclimation period, fish were fed commercial feed (T2-2 Royal Optime, Skretting; proximate composition: 44% crude protein; 21% crude fat; 6.9% crude ash; 2.9% crude fiber) to apparent satiation.

Before the start of the nutritional trial, parrs (n = 696) were gently anesthetized (50 mg/l tricaine methanesulfonate, MS-222, Sigma-Aldrich, Madrid, Spain) and individually measured in body weight (BW) and standard length (SL) to the nearest 0.1 g and 1 mm, respectively. Fish measuring 55.0 ± 0.1 g and 16.2 ± 0.2 mm in BW and SL, respectively, were distributed homogeneously among the 12 experimental tanks (n = 58 fish per tank; 6 replicate tanks per experimental diet). During the trial that lasted 133 days, fish were fed at the daily rate of 3.0% based on the stocked biomass by means of automatic feeders (ARVO-TEC T Drum 2000; ARVO-TEC, Finland). Feed ration was evenly distributed in six meals per day from 07:00 to 17:00 h and regularly adjusted by means of intermediate samplings along the trial according to the stocked biomass in order to guarantee apparent satiation.

The experiment consisted of two different periods with regard to the smoltification process of Atlantic salmon juveniles. During the parr phase (47 days; December 19–February 4), water temperature and pH (pH meter 507; Crison Instruments, Barcelona, Spain), salinity (MASTER-20T; ATAGO Co., Ltd., Tokyo, Japan), and dissolved oxygen (OXI330; Crison Instruments) were 12.2 ± 1.0°C, 7.4 ± 0.3, and 9.4 ± 0.8 mg/l (mean ± SD), respectively (**Supplementary Figure 1**). The water flow rate in experimental tanks was maintained at approximately 9.0 l/min (open-flow system), which guaranteed two full tanks’ water renewal per hour. The photoperiod was 8 h light: 16 h darkness.

Smoltification started on February 5 and lasted 10 days. During this period, water salinity was increased progressively at *ca*. 3 ppt per day until reaching 35 ppt according to SARL SALMO recommendations. The water temperature, pH, and oxygen levels during this period were 12 ± 0.1°C, 7.4 ± 0.3, and 9.6 ± 0.2 mg/l (**Supplementary Figure 1**). The photoperiod during the smoltification period was 24 h light, 0 h darkness. Once fish were transferred to seawater on February 14, the water quality and temperature were maintained by means of a water recirculation system (IRTAmar^®^; Spain) that maintained adequate water quality through UV, biological, and mechanical filtration. The water quality parameters during the rest of the trial were 12.1 ± 0.2°C, 7.4 ± 0.3, and 9.5 ± 0.2 mg/l. Ammonia and nitrite were ≤0.07 and 0.14 mg/l, respectively. Ammonia and nitrites were measured twice per week by means of a portable spectrophotometer (Lovibond MD600, Tintometer GmbH, Germany) using the VARIO Ammonia Salicylate F10 mL (Tintometer GmbH, Germany) and NitriVer^®^ 3 Nitrite Reagent (Permachem^®^ Reagent, Hach Lange, GmbH) assays. The photoperiod during the smolt stage was 24 h light: 0 h darkness. The illumination system for the smolt phase consisted of a led illumination system (Celer, Spain) with a light temperature of 4,000 K and light intensity of 1,540 lumens. At the end of the trial, all fish were netted, anaesthetized with MS-222 as previously described, and individually weighted.

### 2.3 Humoral Immune Parameters

After fish were measured, blood (*ca.* 3 ml) was taken from anaesthetized fish (n = 3 fish per tank) by caudal puncture using heparinized vacutainers with 21 G needles (BD Vacutainer^®^ containing lithium heparin 68 IU) and immediately centrifuged (3,000 *× g* for 15 min at 4°C) to separate plasma.

#### 2.3.1 Peroxidase Activity

The peroxidase activity in plasma samples was measured according to Quade and Roth (25). Samples without plasma were used as blanks. Plates were read at λ = 450 nm in a plate reader (SPECTROstar Nano, BMG LABTECH, Ortenberg, Germany). The peroxidase activity present in each sample was expressed as units/mL.

#### 2.3.2 Protease Activity

The protease activity of plasma was quantified using the azocasein hydrolysis assay (26). Aliquots of 10 μl of plasma were incubated overnight at RT and in agitation with 100 μl of ammonium bicarbonate buffer and 125 μl of 2% azocasein (Sigma-Aldrich) in sterile Eppendorfs. The reaction was stopped by adding 250 μl of 10% trichloroacetic acid (TCA). The mixtures were centrifuged (6,000 *× g* 5 min), 100 μl of the supernatants transferred to a flat-bottomed 96-well plate, and 100 μl of 1 N NaOH added. Optical density was read at λ = 450 nm using a plate reader. Plasma was replaced by trypsin (5 mg/ml, Sigma-Aldrich) for the positive controls (100% of protease activity) or by ammonium bicarbonate buffer for the negative controls (0% of protease activity). The activity for each sample was expressed as % protease activity in relation to the controls.

#### 2.3.3 Antiprotease Activity

The antiprotease activity of plasma was determined by the ability of plasma to inhibit trypsin activity (27). Briefly, 10 μl of plasma samples were incubated (10 min, 22°C) with the same volume of standard trypsin solution (5 mg/ml). After adding 100 μl of 100 mM ammonium bicarbonate buffer and 125 μl of buffer containing 2% azocasein (Sigma-Aldrich), samples were incubated (2 h, 30°C) and, following the addition of 250 μl of 10% TCA, a new incubation (30 min, 30°C) was done. The mixture was then centrifuged (1,500 *× g* 10 min) being the supernatants transferred to a 96-well plate in triplicate containing 100 μl well^−1^ of 1 N NaOH, and the optical density read at λ = 450 nm using a plate reader. For positive control, buffer replaced plasma and trypsin, and for negative control, buffer replaced the plasma. Activity for each sample was expressed as % antiprotease activity in relation to the controls.

#### 2.3.4 Lysozyme Activity

Plasma lysozyme activity was measured by using a turbidimetric method (28) with some modifications. Samples of 20 μl of plasma diluted 1:10 with 0.04 M NaH_2_PO_4_–Na_2_HPO_4_ buffer, pH 6.2, were placed in a flat-bottomed 96-well plate. To each well, 200 μl of freeze-dried *Micrococcus lysodeikticus* in the above buffer (0.3 mg/ml, Sigma-Aldrich) was added as lysozyme substrate. The reduction in absorbance at 450 nm was measured over 15 min at 3-min intervals at RT in a plate reader. One unit of lysozyme activity was defined as a reduction in absorbance of 0.001 per min. The units of lysozyme present in plasma were obtained from a standard curve made with hen egg white lysozyme (HEWL, Sigma-Aldrich). The lysozyme activity for each sample was expressed as μg/mL of hen egg white lysozyme eq. activity.

#### 2.3.5 Bactericidal Activity

Two pathogenic bacteria for fish (*Vibrio anguillarum* and *Vibrio harveyi*) were used in the bactericidal assays. All bacterial strains were grown from 1 ml of stock culture that had been previously frozen at −80°C. The two bacteria were cultured for 48 h at 25°C in Tryptic Soy Agar (TSA, Difco Laboratories) and then inoculated in Tryptic Soy Broth (TSB, Difco Laboratories), both supplemented with NaCl to a final concentration of 1% (w/v). Bacteria in the TSB medium were then cultured at the same temperature, with continuous shaking (100 rpm) for 24 h. Exponentially growing bacteria were resuspended in sterile PBS and adjusted to 10^8^ colony forming units (CFU) per mL.

Bactericidal activity was determined following the method of Stevens et al. (29) using the MTT assay, which is based on the reduction of the yellow soluble tetrazolium salt (3-(4,5-dimethylthiazol-2-yl)-2,5-diphenyltetrazolium bromide) (MTT, Sigma-Aldrich) into a blue, insoluble formazan product by the mitochondrial succinate dehydrogenase (30). Samples of 20 μl of plasma were added in a flat-bottomed 96-well plate. PBS was added to some wells instead of the samples and served as a positive control. Aliquots of 20 μl of the bacteria previously cultured were added, and the plates were incubated for 5 h at 25°C. After that, 25 μl of MTT (1 mg/ml) was added to each well and the plates were newly incubated for 10 min at 25°C to allow the formation of formazan. Plates were then centrifuged (2,000 g, 10 min), with the precipitates dissolved in 200 μl of DMSO and transferred to a new flat-bottom 96-well plate. The absorbance of the dissolved formazan was measured at 570 nm in a plate reader. Bactericidal activity was expressed as the percentage of no viable bacteria, calculated as the difference between absorbance of bacteria surviving compared to the absorbance of bacteria from positive controls (100%).

### 2.4 Bacterial Challenge

In order to investigate the immunomodulatory properties of the phytogenic compounds against bacterial infection, an experimental bacterial challenge with the strain IRTA-17-44 of *A*. *salmonicida* subsp. *salmonicida* (courtesy of HIPRA culture collection, coded: AS8074) was performed at the end of the nutritional trial. Bacterial suspensions of the selected strain were prepared from a stock stored in glycerol at -80°C. The inoculum was grown in TSA at 23.0 ± 1.0°C for 48 h. The bacterial inoculum was prepared to an OD of λ = 550 nm of 1.2, corresponding to a density of 10^8^ CFU/ml previously established by serial dilutions and plate counting. The bacterial suspension was 10-fold serially diluted in sterile PBS, to prepare the desired inoculum, which was confirmed by CFU’s plate counting. Prior to the challenge trial, an *A*. *salmonicida* (IRTA-17-44) lethal dose of 50% (LD_50_) was determined for the experimental conditions to be assayed. For this purpose, 30 control Atlantic salmon were injected intraperitoneally (IP) with 0.2 ml of three concentrations of *A*. *salmonicida* inoculum, 10^6^, 10^7^, and 10^8^ CFU/mL (10 fish injected with each inoculum concentration). Ten additional fish were injected with PBS as methodological control. The concentration of 10^7^ CFU/mL was established as the nearest LD_50_ (data not shown).

For the challenge trial, 32 Atlantic salmon smolts (BW = 194.0 ± 29.1 g) per each dietary treatment were randomly distributed (https://www.randomizer.org) into quadruplicate tanks (four tanks per dietary treatment), with eight fish per tank (stocking density = 14–16 kg m^-3^). During the acclimation period (5 days), fish were fed *ad libitum* with the same experimental diets used in the nutritional assay. After acclimation, fish were anaesthetized and IP injected with 0.2 ml of 10^7^ CFU/ml of *A*. *salmonicida* (IRTA-17-44).

Both the establishment of the *A*. *salmonicida* LD_50_ and the challenge trial were performed at IRTA’s biosafety challenge room, in 32 cylindrical tanks (100 l) connected to a RAS unit (IRTAmar^®^) equipped with real-time control of oxygen and temperature, mechanical filtration, biofiltration, and ultraviolet disinfection of the water. The outflow water was chlorinated, followed by ozone treatment before being discharged. The water quality conditions in terms of temperature and salinity were 13.1 ± 1.1°C and 32.3 ± 0.4 ppt, respectively.

Fish mortality occurring after 12 h post-injection (hpi) was considered to be induced by the pathogen infection rather than handling stress, since no casualties were found in the control group injected with PBS. During the duration of the challenge (12 days), smolts were supervised every 2 h, six times per day, including weekends. In order to avoid unnecessary suffering, when the animals became moribund (i.e., loss of equilibrium, swollen abdomens, hemorrhaging in the anal area, and erratic swimming), they were euthanized with an overdose of MS-222. At the end of the experiment, all the remaining fish were sacrificed following the same procedure.

Confirmation of cause of death was determined by the recovery of the bacteria from all moribund animals, followed by specific PCR using *A*. *salmonicida* specific primers (31). For this purpose, animals were aseptically opened and a tissue sample of HK was taken and plated on TSA, incubated at 23°C for 72 h. Bacterial colonies were collected from the agar using sterile toothpicks and placed into 200 μl of DNA extraction lysis buffer containing proteinase K, and extractions performed following the manufacturer’s protocol (DNeasy Blood and Tissue Kit, Qiagen, Spain). Extracted DNA was evaluated by spectrophotometry to determine the purity and concentration prior to PCR analysis. Amplification was performed in 25-μl reactions containing Taq polymerase buffer (1×), 0.5 U of Taq polymerase, MgCl_2_ (2 mM), dNTPs (900 μM), and 1 μM of each primer specific for *A*. *salmonicida* [forward primer: 5′-CGGTTTTGGCGCAGTGACG-3′ and reverse primer: 5′-AGGCGCTCGGGTTGGCTATCT-3′; Beaz-Hidalgo et al. (31)]. The conditions for amplification were as follows: initial denaturation of template DNA at 95°C for 10 min, followed by 30 cycles of 1 min at 92°C, 1 min at 55°C, and 1 min at 72°C with a final extension step of 5 min at 72°C. Reactions lacking DNA, and containing genomic DNA of *A*. *salmonicida*, were used as negative and positive controls, respectively. PCR products were separated on a 1.2% (w/v) agarose gel and visualized using ethidium bromide staining. The presence of bands with a size of 422 bp was considered as a positive result.

### 2.5 Transcriptional Analysis

#### 2.5.1 RNA Isolation and Quality Control

**Figure 1:**
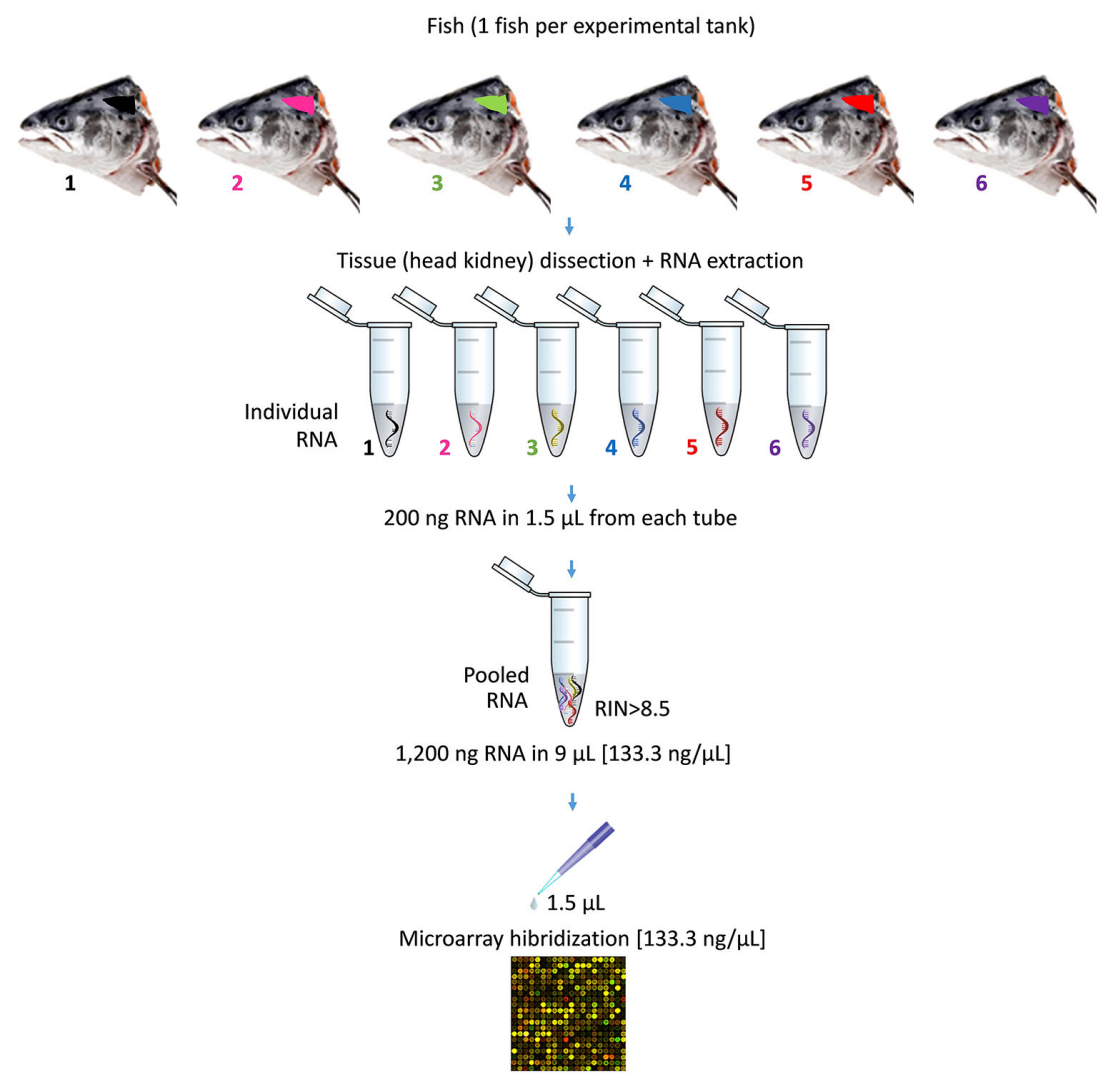
Representation of pooling procedure for microarray hybridization of RNA samples. The diagram describes the pooling of one of the three pools used for each experimental diet. In particular, six individual samples of RNA were obtained from the head kidney of six Atlantic salmon (*S. salar*). Each of sampled fish came from one of the six experimental tank replicates per diet. The pooled RNA was prepared using 200 ng of each individual RNA in 1.5 µl (final volume of the pooled RNA = 9 µl; RNA concentration of the pooled RNA = 133.33 ng/µl). Prior to microarray hybridization, samples of pooled RNA were checked for quality and integrity. Then, pooled RNA were hybridized (1.5 µl; final RNA concentration = 133.33 ng/µl).

At the end of the nutritional assay, the total RNA from the HK of individual fish (n = 18 fish per dietary treatment) was extracted using TRI reagent (Sigma-Aldrich, Sant Louis, MO, USA), according to the manufacturer’s instructions. Total RNA concentration and purity were quantified using a NanoDrop-2000^®^ spectrophotometer (Thermo Scientific, USA) and stored at -80°C for further analysis. Samples were diluted to 133.33 ng/µl concentration and checked for integrity using an Agilent 2100 Bioanalyzer (Agilent Technologies, Spain). All the samples used in this study were selected by the criteria of a RNA Integrity Number (RIN) value >8.5. Three pooled samples for each diet were used for microarray hybridization. Each pool consists in n = 1 fish from each replicate tank per treatment (n = 18 fish per diet, total N = 36 fish) (**Figure 1**). The information regarding individual variability was lost with this choice.

#### 2.5.2 Microarray Design and Analysis

Transcriptional analysis was carried out using the custom-commercial *Salmo salar* oligonucleotide microarray platform (AMADID 084881; Gene Expression Omnibus (GEO) access number: GPL28080, Agilent Technologies; USA). Data presented in this manuscript are available in the GEO accession number GSE179142.

The transcriptomic analysis of HK samples from Atlantic salmon smolts was conducted as described by Reyes-López et al. (32). One-color microarray was carried out according to the manufacturer’s protocols. In brief, 200 ng of total RNA from each pooled samples was reverse transcribed with Agilent One Color RNA Spike-In Kit (Agilent Technologies, USA). Then, total RNA was used as template for Cyanine-3 (Cy3)-labeled cRNA synthesis and amplification with the Quick Amp Labeling Kit (Agilent Technologies). cRNA samples were purified using the RNeasy Micro Kit (Qiagen). Dye incorporation and cRNA yield were checked with the NanoDrop ND-2000^®^ spectrophotometer. Then, 1.5 mg of Cy3-labeled cRNA with specific activity >6.0 pmol Cy3 mg-1 cRNA was fragmented at 60°C for 30 min, and then the samples were mixed with hybridization buffer and hybridized to the array (ID 084881, Agilent Technologies) at 65°C for 17 h using the Gene Expression Hybridization Kit (Agilent Technologies). The microarray washes were conducted as recommended by the manufacturer using Gene Expression Wash Buffers (Agilent Technologies) and stabilization and drying solutions (Agilent Technologies). Microarray slides were scanned with an Agilent Technologies Scanner (model G2505B); spot intensities and other quality control features were extracted with Agilent’s Feature Extraction software version 10.4.0.0 (Agilent Technologies). Quality reports were checked for each array. The identification of differential expressed genes was done, as described elsewhere (33). In brief, the bioinformatic package STARS (Nofima, Norway) was used for data processing and mining (34). After filtration of low-quality spots, Lowess normalization of log2-expression ratios (ER) was performed. The differentially expressed genes (DEGs) were selected by difference between the control and the experimental diet following an unpaired *t*-test. Expression values with a *p*-value < 0.05 were considered statistically significant.

#### 2.5.3 Functional Network Analyses: Interactomes

The complete map of interactions that can occur in a living organism (interactome) was obtained from the DEGs obtained in the microarray-based transcriptomic analysis (transcripteractome). The analysis was performed as described elsewhere (32). In brief, the Search Tool for the Retrieval of Interacting Genes (STRING) public repository version 10.0 (https://string-db.org) was used (35). The protein–protein interaction (PPI) network for the differentially expressed genes was conducted with a high-confidence interaction score (value = 0.4). The mechanisms of response in which DEGs are involved were obtained from a comparative analysis based on *Homo sapiens* as a reference organism in order to extract the maximum information currently available. Thus, an *H. sapiens* acronym was assigned based on *S. salar* transcript annotation using UniProt (36) and GeneCards (37) databases. For those genes with no annotation match in salmon, an orthologue *H*. *sapiens* Entrez Gene was assigned based on sequence homology. To do it, we selected the best tBlastX (NCBI) hit for the DEG query sequence for *S*. *salar* and the human transcriptome database. We only consider those matches with at least E value ≤1e^-10^. The UniProt and GeneCards databases were used to confirm match of the gene acronym tag between both species. Gene ontology (GO) pathway enrichment analysis for biological processes (GO_BiologicalProcess-EBI-UniProt-GOA-ACAP-ARAP_10.11.2020_00h00) was obtained using the ClueGO v2.5.7 (38) app through the Cytoscape 3.8.2 (39) platform. The statistical analysis used was Enrichment/Depletion (two-sided hypergeometric test) with a *p-*value cutoff = 0.05 and corrected by Benjamini–Hochberg; a GO Fusion was performed to avoid redundant terms with a kappa score threshold = 0.4 in order to propose more stringent GO terms associated with the mechanism of response for the experimental diet incorporating the tested phytogenic. In addition, grouping of the GO terms was conducted when the sharing-group percentage was above 50, a *p*-value of < 0.05 was considered as significant. The statistically significant GOs obtained from the enrichment analysis were assigned to each one of the nodes represented in the functional network. The nodes classified in different clusters according to their functionality were represented with ClueGO v2.5.7.

### 2.6 Ethics Statement

All animal experimental procedures complied with the Guiding Principles for Biomedical Research Involving Animals (EU2010/63) and the guidelines of the Spanish laws (law 32/2007 and RD 1201/2015) and were authorized by the Ethical Committee of the Institute for Research and Technology in Food and Agriculture (IRTA, Spain) for the use of laboratory animals (FUE-2020-01314717).

### 2.7 Statistics

Growth performance was compared between groups with a *t*-test (*p* < 0.05). For the challenge trial, the mortality was registered in both experimental diets and data were represented using Kaplan–Meier mortality curves (40). The percent survival was calculated using the Mantel–Cox log-rank test. To construct the hierarchical heatmap, the Heatmapper server was used (41). Results related to the immune parameters were expressed as means ± standard error of mean (SEM). The normality of the variables was confirmed by the Shapiro–Wilk test while the homogeneity of variance was confirmed by the Levene test. Data were statistically analyzed by Student’s *t*-test (*p* < 0.05) to determine significant differences between experimental groups. All the data were analyzed by the computer application SPSS for Windows^®^ (version 15.0, SPSS Inc., Chicago, USA). All the determinations were performed in triplicates.

## 3 Results

### 3.1 Growth Performance

After the 133-day of nutritional trial, no significant differences were observed in growth (252.3 ± 9.2 g *vs*. 240.2 ± 19.3 g) and Fulton’s conditions factor (K = 1.2 ± 0.2 *vs*. 1.3 ± 0.1) between smolts fed the control diet and diet containing 0.15% AQUOLIVE^®^ (*p* > 0.05), respectively.

### 3.2 Non-Specific Humoral Immune Parameters

**Figure 2:**
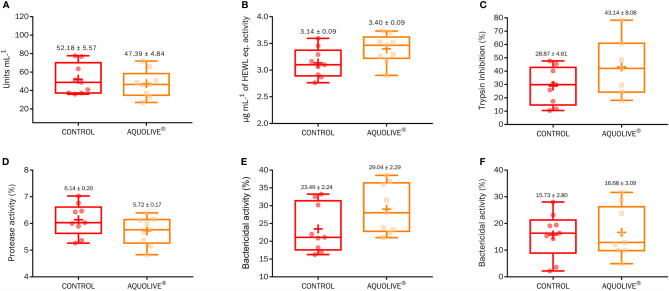
Box-plot of humoral immunity (**A**, peroxidase; **B**, lysozyme; **C**, antiprotease; **D**, protease; **E**, bactericidal activity against *Vibrio anguillarum*; **F**, bactericidal activity against *Vibrio harveyi*) parameters measured in Atlantic salmon (*S. salar*) plasma fed the control diet and diet containing 0.15% AQUOLIVE^®^. Box-plot central lines indicate the median, and the plus symbol indicates the mean of data.

At the end of the feeding trial, there were no significant differences in the humoral immunity (peroxidase, lysozyme, antiprotease, protease, and bactericidal activity) among Atlantic salmon smolts fed both diets (**Figure 2**; *p* > 0.05).

### 3.3 Head Kidney Transcriptomic and Microarrays

**Figure 3:**
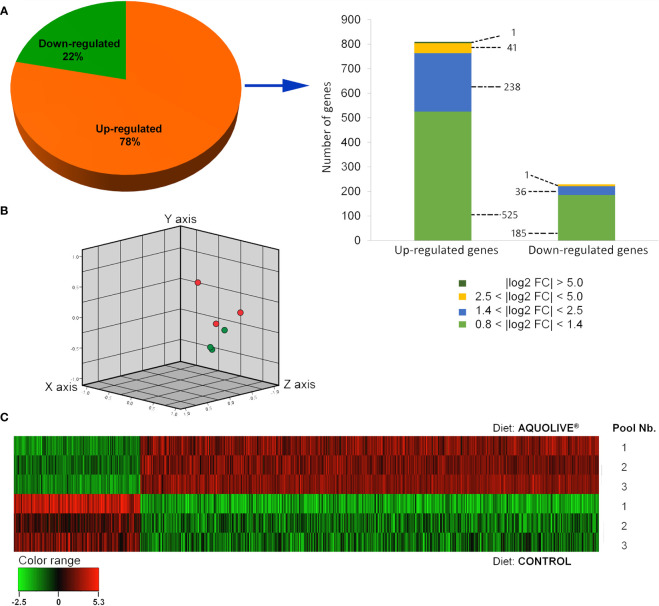
Differential expression analysis of the Atlantic salmon (*S*. *salar*) head kidney transcriptomic response to AQUOLIVE^®^ diet. **(A)** Distribution (pie chart) of the differential expressed genes (DEGs) obtained from the microarray-based transcriptomic analysis response fed a diet supplemented with a phytochemicals from olive fruit. Absolute log2 fold-change (|log2 FC|) indicates the magnitude interval of response. **(B)** Principal component analysis (PCA) of the DEGs for the Atlantic salmon head kidney in response to the control (red node) and phytogenic-supplemented diet (green node). **(C)** Hierarchical clustering of the Atlantic salmon head kidney transcriptomic response for the control and AQUOLIVE^®^ diet, based in similitude patterns of the DEGs detected from three sample pools per dietary group. Data of the six microarrays are depicted. The normalized intensity values (log2) obtained for each microarray analyzed for control (replicates 1, 2, and 3) and AQUOLIVE^®^ group (replicate 1, 2, and 3) are shown.

In order to determine the modulatory effect of the dietary supplementation with phytochemicals obtained from olive fruit upon the Atlantic salmon HK transcriptome, a microarray-based transcriptomic analysis was conducted (**Figure 3**). A total number of 1,027 DEGs were found when comparing the transcriptomic profiling of the HK from Atlantic salmon fed the control and AQUOLIVE^®^ diets (*p* < 0.05; **Supplementary Table 1**). In the case of upregulated genes, most of the transcripts (525) were identified in the 0.8 < log2 absolute fold change (|log2 FC|) < 1.4 interval. Then, 238 transcripts were identified in the 1.4 < |log2 FC| < 2.5 interval, 41 transcripts in the 2.5 < |log2 FC| < 5.0, and only one single gene in the |log2 FC| > 5.0. For the downregulated genes, 185 transcripts were identified in the 0.8 < |log2 FC| < 1.4 interval. Thirty-six other transcripts were grouped in the 1.4 < |log2 FC| < 2.5 interval, meanwhile only 1 DEG was included in the 2.5 < |log2 FC| < 5.0 expression interval. The detailed analysis of the gene absolute log2 fold change (|log2 FC|) revealed that genes were mostly upregulated in fish fed the AQUOLIVE^®^ diet (78.4% of DEGs), while its gene modulation was moderate in terms of FC intensity (**Figure 3A**). Results from the three-principal component of the PCA analysis revealed a segregation pattern among dietary treatments pools. Differential gene expression patterns between the control and AQUOLIVE^®^ groups are shown in **Figure 3B**. In addition, when representing DEGs intensity values from the pooled samples, a common segregation among profiles was observed in the hierarchical clustering heatmap for the HK transcriptomic response between AQUOLIVE^®^ and control diet (*p* < 0.05; **Figure 3C**).

### 3.4 Enrichment Analyses

**Figure 4:**
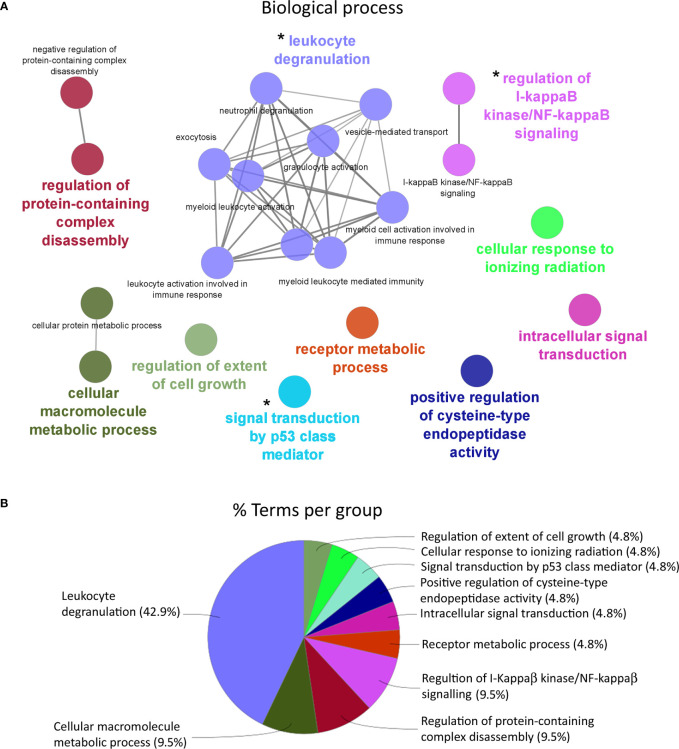
Functional enrichment network analysis for biological processes based on the total number of differential expressed genes (DEGs) in the head kidney of Atlantic salmon (*S. salar*) fed with AQUOLIVE^®^ diet. **(A)** Different biological processes represented in head kidney transcripteractome in response to the phytochemicals obtained from the olive fruit. Each color indicates a cluster of closely related biological processes. The colored biological term denominates the leading group term. The lines into the cluster indicated the close relationship between biological processes. The asterisks (*) indicate the three main clusters related to immune response pathways regulated by the feed additive. **(B)** Overview chart with functional groups including specific terms for DEGs of the Atlantic salmon head kidney transcriptomic response to AQUOLIVE^®^-supplemented diet, distribution of the biological processes according to their percentage of representation upon the total enriched terms is shown.

An enrichment analysis was carried out in order to determine those biological processes represented for the differentially expressed genes in HK response (**Figure 4**). For the enriched biological processes in HK of the Atlantic salmon (**Figure 4A**) fed with AQUOLIVE^®^, 10 representative groups were identified in the transcripteractome: “regulation of extent of cell growth” (4.76%; GO:0061387), “cellular response to ionizing radiation” (4.76%; GO:0071479), “signal transduction by p53 class mediator” (4.76%; GO:0072331), “positive regulation of cysteine-type endopeptidase activity” (4.76%; GO:2001056), “intracellular signal transduction” (4.76%; GO:0035556), “receptor metabolic process” (4.76%; GO:0043112), “regulation of i-kappaB kinase/NF-kappaB signaling” (9.52%; GO:0043122), “regulation of protein-containing complex disassembly” (9.52%; GO:0043244), “cellular macromolecule metabolic process” (9.52%; GO:0044260), and “leukocyte degranulation” (42.86%; GO:0043299) (**Figure 4B**).

**Figure 5:**
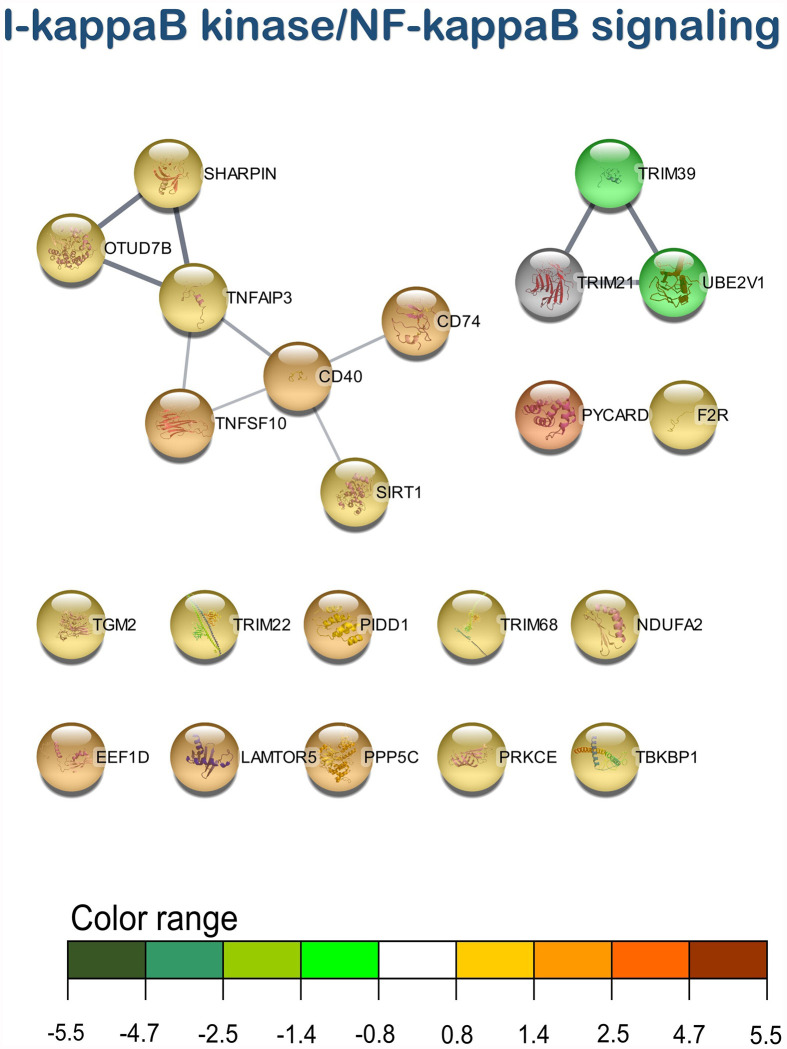
I-kappaB kinase/NF-kappaB signaling transcripteractome of the differentially expressed genes (DEGs) related to the immune pathway in the head kidney of juvenile Atlantic salmon (*S*. *salar*) fed the AQUOLIVE^®^ diet (see also **Supplementary Table 2**). Color range indicates the modulation in terms of log2 fold change (log2 FC) intensity of each node.

**Figure 6:**
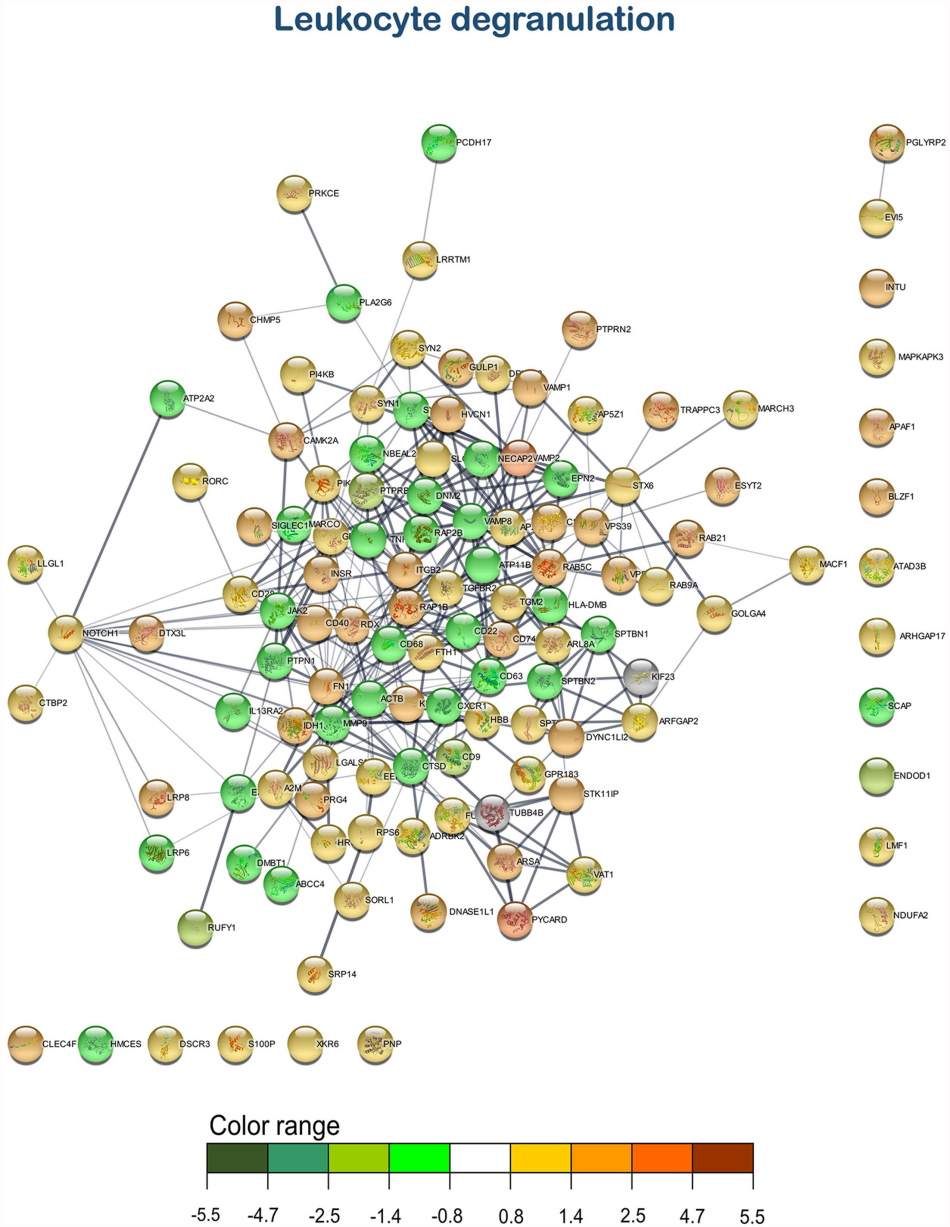
Leukocyte degranulation transcripteractome of the differentially expressed genes (DEGs) related to the immune pathway in the head kidney of juvenile Atlantic salmon (*S*. *salar*) fed the AQUOLIVE^®^ diet (see also **Supplementary Table 3**). Color range indicates the modulation in terms of log2 fold change (log2 FC) intensity of each node.

**Figure 7:**
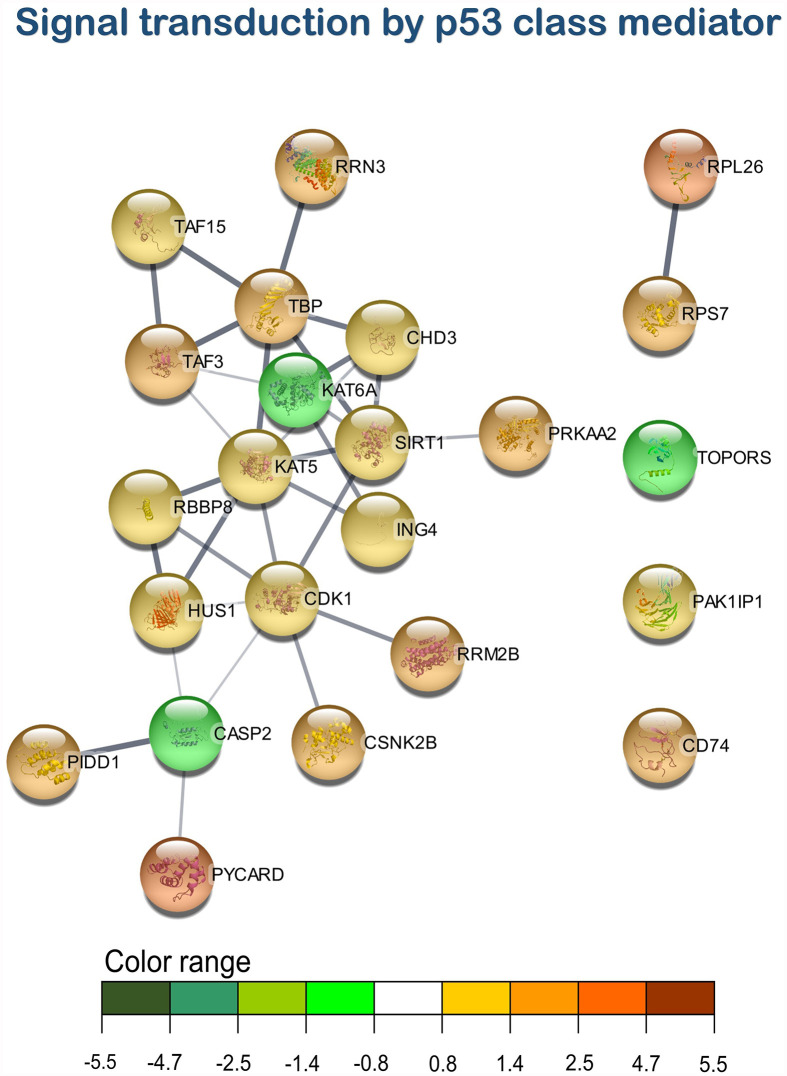
Signal transduction by p53 class mediator transcripteractome of the differentially expressed genes (DEGs) related to the immune pathway in the head kidney of juvenile Atlantic salmon (*S*. *salar*) fed the AQUOLIVE^®^ diet (see also **Supplementary Table 4**). Color range indicated the modulation in terms of log2 fold change (log2 FC) intensity of each node.

According to the enrichment results, three main representative clusters of genes related to immunity were identified in the transcripteractome among the totality of biological processes obtained from the enrichment analysis: (1) “i-kappaB kinase/NF-kappaB signaling” (**Figure 5**), “leukocyte degranulation” (**Figure 6**), and “signal transduction by p53 class mediator” (**Figure 7**). **Table 2** summarizes the most relevant DEGs in terms of FC in fish fed the AQUOLIVE^®^ diet in relation to the abovementioned biological processes.

**Table 2:** List of the most relevant DEGs related to the three main representative biological processes identified by the transcripteractome in fish fed the AQUOLIVE^®^ diet.

Gene description	Gene acronym	FC (log2)	*p*-value
Ribosomal protein L26	*rpl26*	3.43	0.02429
Vesicle-associated membrane protein 2	*vamp2*	3.40	0.01714
PYD and CARD domain containing	*pycard*	2.72	0.00004
RAB21, member RAS oncogene family	*rab21*	2.35	0.00369
RAB5B, member RAS oncogene family, b	*rab5b*	2.20	0.04218
RAS related protein 1b	*rap1b*	2.17	0.03414
CD40 molecule	*cd40*	2.02	0.00121
TNF superfamily member 10	*tnfsf10*	1.85	0.03433
P53-induced death domain protein 1	*pidd1*	1.66	0.01492
CD74 molecule	*cd74*	1.63	0.00496
RAB9A, member RAS oncogene family, b	*rab9a*	1.10	0.00110
CD28 molecule	*cd28*	1.08	0.03644
TNF alpha-induced protein 3	*tnfaip3*	0.88	0.03090
Alpha-2-macroglobulin	*a2m*	0.80	0.04309
CD68 molecule	*cd68*	-0.86	0.00752
CD9 molecule	*cd9*	-0.89	0.02258
CD63 molecule	*cd63*	-0.95	0.01869
CD22 molecule	*cd22*	-1.02	0.01087
Caspase 2	*casp-2*	-1.31	0.03100
Protein tyrosine phosphatase receptor type b	*ptprb*	-1.81	0.00296
Endonuclease domain-containing 1	*endod1*	-1.86	0.03239

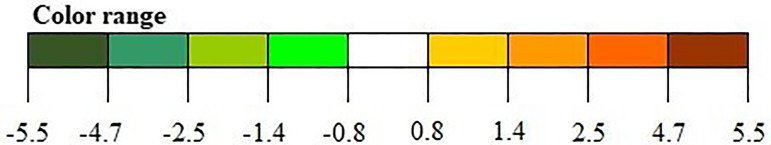

As mentioned above, three main clusters regarding the dietary regulation of biological processes related to HK immunity were identified. For the cluster of “regulation of i-kappaB kinase/NF-kappaB signaling”, two nodes were observed including “I-kappaB kinase/NF-kappaB signaling” (GO:0007249; 19 upregulated genes; 2 downregulated genes) and “regulation of I-kappaB kinase/NF-kappaB signaling” (GO:0043122; 17 upregulated genes; 2 downregulated genes). In the “leukocyte degranulation” cluster, the other nine nodes were identified including “myeloid leukocyte activation” (GO:0002274; 29 upregulated genes; 13 downregulated genes), “leukocyte activation involved in immune response” (GO:0002366; 34 upregulated genes; 14 downregulated genes), “myeloid cell activation involved in immune response” (GO:0002275; 27 upregulated genes; 12 downregulated genes), “exocytosis” (GO:0006887; 38 upregulated genes; 18 downregulated genes), “granulocyte activation” (GO:0036230; 26 upregulated genes; 11 downregulated genes), “leukocyte degranulation” (GO:0043299; 26 upregulated genes; 12 downregulated genes) “neutrophil degranulation” (GO:0043312; 25 upregulated genes; 11 downregulated genes), and “vesicle-mediated transport” (GO:0016192; 79 upregulated genes; 35 downregulated genes). Lastly, one single-node cluster was identified including “signal transduction by p53 class mediator” (GO:0072331; 20 upregulated genes; 3 downregulated genes).

### 3.5 *In Vivo* Bacterial Challenge Test

**Figure 8:**
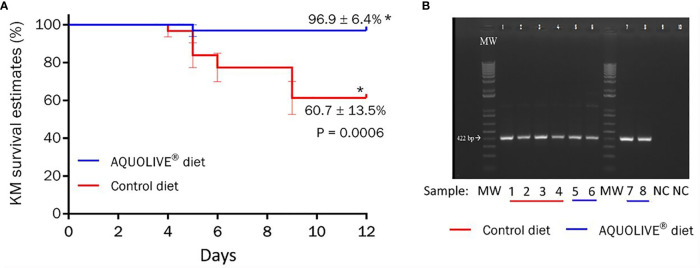
Results of the bacterial challenge conducted in Atlantic salmon smolts intraperitoneally injected with 1 × 10^7^ CFU/ml of *A. salmonicida.* **(A)** Kaplan–Meier (KM) survival curves (%) for Atlantic salmon smolts intraperitoneally injected with *A. salmonicida* (1 × 10^7^ CFU/ml) during the 12-day challenge trial period. Data correspond to the mean ± standard error (four replicates tanks per experimental diet; n = 8 fish per tank). The asterik (*) indicates statistically significant differences among dietary treatments (t-test; p < 0.05). **(B)** Specific PCR of bacterial colonies recovered from smears of head kidney from moribund Atlantic salmon smolts during the bacterial challenge test with *A*. *salmonicida*. MW = molecular weight standard; lanes 1–6 are samples recovered from moribund fish (1–3: control diet; 4–6: AQUOLIVE^®^ diet); lanes 7–8 are positive control genomic DNA from *A. salmonicida*; lanes 9–10 are negative control lacking template DNA.

During the *in vivo* bacterial challenge test with *A*. *salmonicida* (intraperitoneal injection: 1 × 10^7^ CFU/ml), mortality in smolts was observed between 4 and 9 days post-injection (**Figure 8**). The Kaplan–Meier survival curves showed significant differences in terms of Atlantic salmon smolt survival depending on the dietary condition considered (**Figure 8A**; *p* < 0.05). In particular, smolts fed the AQUOLIVE^®^ diet showed higher survival rates (96.9 ± 6.4%, mean ± standard deviation) in comparison to their congeners fed the control diet (60.7 ± 13.5%). To confirm the cause of death, species-specific PCR was performed from bacterial colonies recovered from HK smears of all moribund fish during the bacterial challenge assay. Confluent pure bacterial growth was obtained from all animals, from which *A*. *salmonicida* was confirmed in all cases by means of PCR as shown in **Figure 8B**.

## 4 Discussion

The market for sustainable products and feed additives is increasingly growing. The number of studies focused on the use of a wide variety of phytogenics as sustainable tools to be implemented in aquaculture production has dramatically increased in the last years. This has been mainly due to phytogenics’ growth-promoting, antimicrobial, immunostimulant, antioxidant, and anti-inflammatory properties (13, 42). In this study, we have evaluated a new phytogenic feed additive rich in triterpenic compounds and polyphenols derived from olive fruit (AQUOLIVE^®^) on the systemic immune response and disease resistance in Atlantic salmon smolts. In this context, a total number of 1,027 DEGs (805 up- and 222 downregulated) were found when comparing the transcriptomic profiling of the HK from fish fed the control and AQUOLIVE^®^ diets. Moreover, the bacterial challenge lasted 12 days at the end of the assay, showing that the cumulative survival was higher in fish fed the AQUOLIVE^®^ diet (96.9 ± 6.4%) than in fish from the control group (60.7 ± 13.5%).

Previous studies on the inclusion of bioactive compounds derived from the olive industry have been conducted. Particularly, it has been shown that a diet with olive oil bioactive extract rich in triterpenic compounds enhanced the innate immune function and integrity in the intestine of gilthead seabream (*Sparus aurata*) (22). Additionally, a phytogenic with similar bioactive compounds than AQUOLIVE^®^ showed a tightly controlled systemic immune response in an *ex vivo* assay using splenocytes stimulated by lipopolysaccharide (LPS) (43). Regarding the dietary supplementation of olive leaf extracts, Navruz et al. (44) reported that common carp (*Cyprinus carpio*) showed an improved immune response and survival rates against *Edwardsiella tarda*. Similarly, in rainbow trout (*Oncorhynchus mykiss*) feed phytogenic compounds derived from olive leaf extract (OLE) showed an enhancement of the expression of immune-related genes, such as pro-inflammatory cytokines like *tnfα*, *il1-β*, and *il-8*, as well as disease resistance against *Yersinia ruckeri* (45). The abovementioned results are in agreement with the results obtained in our study when Atlantic salmon smolts fed the AQUOLIVE^®^ diet showed higher disease resistance in front of the pathogenic bacteria *A*. *salmonicida* than their congeners fed the control diet.

### 4.1 Transcription Factors

In order to investigate the immunomodulatory properties of the phytogenic tested, the modulation of the transcriptional immune response in the HK of the anadromous fish Atlantic salmon fed AQUOLIVE^®^ diet was evaluated by means of a microarray analysis. This is of special relevance, since in order to achieve a proper immune response, a wide repertoire of biological processes at cellular and molecular levels, including transcription factors, are usually involved, as described in the following. The dietary supplementation of AQUOLIVE^®^ in the HK of Atlantic salmon shows modulation of different biological processes related to transcription factors such as “signal transduction by p53 class mediator” and “i-kappa B kinase/NF-kappa B signaling”, among others. Different studies have evidenced that there is a transcriptional cross talk between nuclear factor κB (NF-κB) and p53 (46, 47). In particular, NF-κB may be considered as a transcriptional regulator of p53 and *vice versa*. In fact, NF-κB was found to be able to recognize κB sites on the p53 promoter and thereby activate its expression (47). p53 is part of the innate and adaptive immune system, as well as detect DNA damage, repair, and recombination, besides playing an important role in infectious diseases, killing, and limiting viral and bacterial replication (48). In line with this, it has been shown in different fish species that p53 is an important mediator of innate antiviral and antibacterial immunity (49–51). On the other hand, the NF-κB pathway is well known as a central mediator in the regulation of several cytokines, chemokines, antimicrobial peptides, and interferon-stimulated genes, playing a critical role in regulating the survival, activation, and differentiation of innate and adaptive immune cells (52, 53). Moreover, it has been demonstrated that upon bacterial infection, the cytoplasmic NF-κB is rapidly activated and translocated into the nucleus to stimulate the expression of antimicrobial peptides fighting against pathogenic organisms (54). The gene coding for the P53-induced death domain protein (*pidd1*) was upregulated in the HK of Atlantic salmon smolts fed the AQUOLIVE^®^-supplemented diet in comparison to their congeners fed the control diet. This gene is reputed for playing an essential role in NF-κB and caspase-2 activation. It has been shown in the literature that PIDD1 expression causes spontaneous activation of caspase-2 and sensitization to apoptosis by genotoxic stimuli (55, 56). In this sense, *casp-2* expression is involved in the regulation of p53 in response to cellular stress and DNA damage to prevent the proliferation and accumulation of damaged or aberrant cells (56). *Casp-2* was significantly downregulated in the HK of fish fed the AQUOLIVE^®^-supplemented diet, thus leading us to a possible homoeostatic scenario. Another gene involved in the abovementioned biological processes that deserves attention is the PYD and CARD Domain Containing (*pycard*), which was upregulated in our samples from the HK of fish fed the AQUOLIVE^®^ diet. PYCARD is a dual regulator in NF-κB activation pathways and plays a distinct role in innate defense systems through the inflammasome (57, 58). This is relevant, since it has been shown that inflammasome activation plays a critical role in activating innate immunity (59). The inflammasome consists of caspase-1 and caspase-5 enzymes, Pycard/Asc, and NAPL1, a pyrin domain-carrying protein, which shares a structural homology with NODs (nucleotide-binding oligomerization domain-like receptors). In the presence of certain stimuli (e.g., a specific pathogen cell-surface proteins), the caspase-1 scaffold within the inflammasome is activated, which induces the inflammatory response (59, 60). Therefore, it might provide us an answer to the increased disease resistance of Atlantic salmon smolts fed with the tested phytogenic and *in vivo* challenged with *A*. *salmonicida*, obtaining a higher survival when compared to fish fed the control diet (60).

### 4.2 Cell Response

The HK is one of the most important organs in fish due to its role in endocrine and hematopoietic functions, and it is a major secondary lymphoid organ in the body (61). Our findings showed that the tested feed additive regulates several biological processes in the HK related to the host’s immunity. In particular, these biological processes were related to innate immune effector key cell functions of vertebrate innate immunity (62), such as “leukocyte activation”, “granulocyte activation”, “neutrophil degranulation”, “exocytosis”, and “vesicle-mediated transport”, among others. In addition, granulocytes are the main phagocytic cells in the HK and are also involved in the innate immunity as antigen-presenting cells (63). Moreover, neutrophils are one of the three types of granulocytes identified in fish (64, 65), whereas neutrophilic granulocytes are the most abundant in salmonids (66). As their main function is arriving first at the site of the infection and having a central role in host tissue protection by killing pathogenic microorganisms and stimulating lymphocytes and other immune cells, neutrophils are an essential part of the innate immune system (67). In addition, under normal conditions, neutrophils are rarely found in tissues since they are recruited from blood and hematopoietic organs. However, fish neutrophils are not so abundantly present in the bloodstream contrarily to mammals, since they are stored in hematopoietic reservoirs, which may be interpreted as a disadvantage for rapid migration and effective resolution of infection and inflammation events (68). In fact, the dietary supplementation of olive extract or similar bioactive compounds has been reported to enhance hematological and other immune parameters in different animal species, such as reducing inflammation and oxidative stress and enhancing the intestinal immune function, among others (20–22, 69, 70). As previously mentioned, “exocytosis” and “vesicle-mediated transport” were also modulated by AQUOLIVE^®^; this is especially relevant since exocytosis is recognized by its important role in the immune response participating in neutrophil function (71). For instance, genes like vesicle-associated membrane protein 2 (*vamp2*) showed an upregulation when compared to the control diet. VAMP2 is known to participate in different cell types, including neutrophils, monocytes, and eosinophils, regulating exocytosis, since it is predominantly in the membrane of secretory vesicles (72, 73). Thus, the membrane densities of VAMP2 correspond to the exocytic potential of the different storage vesicles, strongly suggesting a functional role of this protein in neutrophil degranulation (71). This is of special relevance, since it has been shown that individuals with decreased or missing neutrophil degranulation had higher incidence of bacterial and fungal infections (74). Therefore, an increase in neutrophil degranulation could lead to enhanced disease resistance and reduced mortality rates in individual fish, as occurred in our bacterial challenge (75). Additionally, transcriptional regulation of vesicle-mediated transport by dietary administration of AQUOLIVE^®^ resulted in the positive regulation of several genes encoding the RAB family of GTPases (*rab21*, *rab5b*, *rap1b*, *rab9a*), recognized for participation in the regulation of exocytosis as leading regulators of membrane trafficking and directing inflammation and immune cellular responses (76, 77). In this sense, phenolic compounds from olive tree leaves have been described to regulate vesicle and exocytic processes (78). Therefore, we hypothesize that the machinery implied in the activation of biological processes observed by dietary AQUOLIVE^®^ may be inherent to the activation of processes of secretory protein translocation by vesicles.

### 4.3 Innate and Adaptive Response

The expression of several genes (*cd9*, *cd22*, *cd28*, *cd63*, *cd68*, *cd74*) associated with innate and adaptive immunity was modulated by the AQUOLIVE^®^-supplemented diet as well. For example, the expression of the gene coding for the CD9 molecule was downregulated in the HK of fish fed the AQUOLIVE^®^ diet. CD9 was found to be extensively present in Atlantic salmon IgM^+^ B cells (79), also known to encode tetraspanins, which are key players in the recruitment of leukocytes into inflammation sites and regulation of several steps of the immune response (80). Castro et al. (81) reported that *cd9* transcription in IgM^+^ B lymphocytes was modulated in the presence of bacteria and virus, in particular, *cd9* was downregulated in rainbow trout in response to a virus, thus revealing a role for this molecule in this antigen-specific lymphocyte response. Therefore, the downregulation of this gene in accordance with our results could suggest a migratory capacity of B cells in response to bacterial or viral infection. Furthermore, the downregulation of the CD63 molecule, another tetraspanin, was also modulated by the tested feed additives. Particularly, it was observed that *cd63* levels were downregulated when exposed *in vivo* in response to a virus, suggesting a possible increase of the antigen-presenting capacity of IgM^+^ cells, as suggested by Castro et al. (81). In this way, the tetraspanin family has been shown to play an important role in influencing MHC II antigen presentation and CD4^+^ T cell stimulation (82). Importantly, Petersen et al. (82) showed that a knockdown of CD63 in the B lymphoblastoid cell line may play a role in participating in the modulation of cell-surface-initiated signals, which can trigger exosomal secretion and lead to increased CD4^+^ T cell recognition. Nevertheless, further studies need to be addressed properly to give us the proper meaning of the downregulation of *cd63* regarding the AQUOLIVE^®^-based feed additive in the HK of Atlantic salmon. Additionally, *cd68* was downregulated by the AQUOLIVE^®^-supplemented diet. This gene is a transmembrane protein with a suspected role in phagocytic activities of tissue macrophages, and it has also been found in granules of neutrophils, as well as in certain epithelial cells (83). Von Rhaden et al. (83) have shown that the upregulation of *cd68* in macrophages was involved in the inflammatory response. Under present experimental conditions, the downregulation of *cd68* may indicate a tight control of the inflammatory response. However, only a few studies were carried out on *cd68* in fish. Thus, the exact function in *cd68* with regard to its nutritional regulation by phytogenics is unclear and further studies are needed. On the other hand, *cd28* and *cd74* both were upregulated in the fish fed the AQUOLIVE^®^-supplemented diet. In particular, CD28 is probably the most important fish T cell co-stimulatory receptors, playing a key part in interactions between lymphocytes and antigen-presenting cells (84). Moreover, CD74 plays a specific role as an important component in the functional presentation of MHC class II-restricted antigens and as a cytokine receptor (85). Therefore, our results are in agreement with another transcriptomic study in which virus-challenged Atlantic salmon had increased expression of both *cd28* and *cd74* genes in the experimental group compared to the control group, resulting in increased resistance to pancreas disease caused by salmonid alphavirus, which is a severe contagious disease in farmed Atlantic salmon (86). In this sense, we found evidence for the activation of specific immunity genes such as B and T lymphocyte activity or MHC class II antigen presentation, suggesting the stimulation of the innate and the adaptive immune response as well through the tested feed additives.

### 4.4 Inflammatory Response and Immune Signaling

Genes that are involved in response to tumor necrosis factor (TNF) family members (*cd40*, *tnfsf10*, *tnfaip3*) were also upregulated by the AQUOLIVE^®^ diet. Particularly, the TNF family plays an especially important role in the immune system; many of these molecules are essential in the regulation of B cell biology and B cell-mediated immune responses (87). Interestingly, it has been demonstrated that the TNF receptor superfamily member 5 (*cd40*) is capable of stimulating the non-canonical NF-κB pathway, in addition to playing an essential role for T and B cell cooperation in response to protein antigens (88, 89). TRAIL, also known as TNF superfamily member 10 (*tnfsf10*), was positively modulated by the tested feed additive, and it has been reported to be involved in the immune response, specifically under parasite infections, and B cell differentiation and survival in front of bacterial and viral infections (87, 90). Biswas et al. (91) reported that the upregulation of the *tnfsf10* gene in Japanese pufferfish (*Takifugu rubripes*) indicated a probable role of this gene in inducing apoptosis in virus-infected cells. In addition, TRAIL was recognized as a critical mediator of the p53 response in the apoptotic pathway (92). Last but not least, the tumor necrosis factor alpha-induced protein 3 (*tnfaip3*) was also upregulated by the AQUOLIVE^®^ diet. TNFAIP3 is a zinc finger domain-containing protein, which is recognized to be a negative regulator of NF-κB signaling (93), thereby negatively regulating the transcription of other pro-inflammatory cytokines and, consequently, controlling the inflammatory response. Therefore, the present results suggest a hypothesis that the tested feed additive promoted an immune homeostatic effect.

Our study also revealed that the ribosomal protein L26 (*rpl26*) was upregulated in the HK of fish fed the phytogenic-supplemented diet, which is involved in the abovementioned “signal transduction by p53 class mediator” biological process. This gene is located at the ribosomal subunit interface of the 60S subunit inside the cell (94). Interestingly, several studies have demonstrated the role of the *rpl26* gene as a phagocytosis-activating protein, thus being highly involved in the immune response, since phagocytosis is a major mechanism used to remove pathogens and cell debris (95–97). Furthermore, it has been possible to demonstrate that the *rpl26* gene has a strong ability to bind p53 mRNA and thereby to stimulate p53 translation, as previously indicated (98, 99). In fact, there is also evidence that the aforementioned function of RPL26 as a phagocytosis-activating protein into the cells may be facilitated by the alpha-2-macroglobulin (*α2M*) (100). Interestingly, *α2m* was also upregulated in fish fed dietary AQUOLIVE^®^. Moreover, this immune-related gene is known to be the most widely studied protease inhibitor that mainly functions to maintain body fluid homeostasis and is also involved in acute-phase reactions and defense against pathogens that secrete proteolytic enzymes. In this sense, *α2M* plays an important role in restricting the ability of bacteria to invade and grow during the infective process (101). This may be of particular relevance, since fish fed the AQUOLIVE^®^ diet demonstrated higher survival (96.9 ± 6.4%) in comparison to fish fed the control diet (60.7 ± 13.5%). It has been found that some highly adapted pathogenic bacteria, like *A*. *salmonicida*, can evade the host defense mechanisms producing a highly toxic serine protease, which can resist some antiproteases (102, 103). However, *α2M* has the capacity to inhibit the serine protease of *A*. *salmonicida*, thus reducing susceptibility to furunculosis among salmonids (102–104). These transcriptomic results from the HK of smolts at the end of the nutritional trial are in agreement with different mortality rates observed between experimental groups when challenged with this pathogenic bacterium.

In addition to evaluating by microarray analysis the potential immunomodulatory effects of the tested plant extract used in this study, the authors wanted to extend these possible effects with other parameters (i.e., humoral immune markers). For this purpose, different humoral immune parameters were evaluated in plasma at the end of the nutritional assay. This evaluation of plasmatic immune parameters (peroxidase, protease, antiprotease, lysozyme, and bactericidal activity) revealed no significant immunostimulant effect of the tested feed additive. These results might be supported by the hypothesis that the use of additives does not always have the expected immunological response if fish are not exposed to a real threat (outbreaks of diseases or a bacterial challenge trial) (43, 105), and also to the fact that the unnecessary activation of immune response would affect the energy budget (106), which may potentially affect growth performance. Nevertheless, it should be noted that in the presence of a pathogen *stimulus*, this basal condition was affected and apparently enhanced when we observed at the DEG analysis of fish fed the AQUOLIVE^®^-supplemented diet.

## 5 Conclusions

In summary, analysis of the HK transcriptomic profiling response to a diet supplemented with 0.15% AQUOLIVE^®^ revealed a gene expression profile that favors biological processes particularly related to immunity. This mechanism activates effector leukocytes such as granulocytes, which differentiate into neutrophils, suggesting an innate immune response promoted by the tested functional feed additive in the HK. The immune response promoted by AQUOLIVE^®^ dietary is also supported by the active control of vesicular transport and exocytosis. The overall results of our study highlighted the main biological processes induced by this dietary AQUOLIVE^®^ which might be responsible for the better performance, as shown by lower mortality rates in fish fed this additive when they were challenged with *A*. *salmonicida*. Altogether, this study indicated that the tested feed additive, rich in triterpenic and polyphenolic compounds from *O*. *europaea*, promotes systemic immunity and protects Atlantic salmon smolts against *A*. *salmonicida*. Thus, the combination of current vaccination practices conducted by the industry coupled with the administration of AQUOLIVE^®^ may represent a good strategy against furunculosis. In addition, this phytogenic may be also of interest for other marine species like European sea bass (*Dicentrarchus labrax*) suffering from furunculosis (107). Moreover, these results indicate that these phytogenics may be a promising tool to be implemented in sustainable and environmentally responsible aquaculture industry in the post-antibiotic era.

## Data Availability Statement

The datasets presented in this study can be found in online repositories. The names of the repository/repositories and accession number(s) can be found in the article/**Supplementary Material**.

## Ethics Statement

All animal experimental procedures were complied with the Guiding Principles for Biomedical Research Involving Animals (EU2010/63) and the guidelines of the Spanish laws (law 32/2007 and RD 1201/2015) and authorized by the Ethical Committee of the Institute for Research and Technology in Food and Agriculture (IRTA, Spain) for the use of laboratory animals (FUE-2020-01314717).

## Author Contributions

Conceptualization, EG. Methodology, MDF, EV-V, FER-L, ME, CE. Formal analysis, RS, FER-L, JF, EV-V. Resources, EG. Writing original draft, RS; writing review and editing, MDF, EV-V, RS, FER, EG, LT; visualization, RS, FER-L, EV-V; supervision, EG, EV-V; project administration, EG; funding acquisition, EG. All authors have read and agreed to the published version of the manuscript.

## Funding

This work has been financially supported by the project “AQUOLIVE by NATAC”, funded by the European Union’s Horizon 2020 research and innovation program (Grant Agreement Nb. 830202). FER-L thanks the support of Fondecyt Regular grant (Nb. 1211841; ANID; Government of Chile). RS is supported by a PhD grant from the government of Paraguay (BECAL). JF has been subsidized by the Industrial PhD program of *Generalitat de Catalunya* and TECNOVIT-FARMFAES S.L. (Nb. 2017 DI 017). The funder was not involved in the study design, collection, analysis, interpretation of data, the writing of this article or the decision to submit it for publication. Collaboration between Ibero-American researchers has been done under the framework of the network LARVAplus “Strategies for the development and improvement of fish larvae production in Ibero-America” (117RT0521) funded by the Ibero-American Program of Science and Technology for Development (CYTED, Spain).

## Conflict of Interest

JQ and JP are current NATAC BIOTECH S.L. employers.

The remaining authors declare that the research was conducted in the absence of any commercial or financial relationships that could be construed as a potential conflict of interest.

## Publisher’s Note

All claims expressed in this article are solely those of the authors and do not necessarily represent those of their affiliated organizations, or those of the publisher, the editors and the reviewers. Any product that may be evaluated in this article, or claim that may be made by its manufacturer, is not guaranteed or endorsed by the publisher.
